# Prevalence and factors associated with nontuberculous mycobacteria in non-cystic fibrosis bronchiectasis: a multicenter observational study

**DOI:** 10.1186/s12879-016-1774-x

**Published:** 2016-08-22

**Authors:** L. Máiz, R. Girón, C. Olveira, M. Vendrell, R. Nieto, M. A. Martínez-García

**Affiliations:** 1 Pneumology Service, Chronic Bronchial Infection, Cystic Fibrosis and Bronchiectasis Unit, Hospital Universitario Ramón y Cajal, Carretera de Colmenar Km 9,100, 28034 Madrid, Spain; 2 Pneumology Service, Hospital La Princesa, Institute for Health Research (IP), Hospital Universitario de La Princesa, Madrid, Spain; 3 Pneumology Service, Hospital Universitario Regional de Málaga, Instituto de Biomedicina de Málaga (IBIMA), Málaga University, Málaga, Spain; 4 Bronchiectasis Group (Girona Biomedical Research Institute) IDIBGI, Dr Trueta University Hospital, Girona, Spain; CIBER de Enfermedades Respiratorias (Ciberes CB06/06/0030), Instituto de Salud Carlos III, Madrid, Spain; 5 Pneumology Service, Hospital Universitario y Politécnico La Fe, CIBERes, CIBER de Enfermedades Respiratorias, Valencia, Spain

**Keywords:** Nontuberculous mycobacteria, Disease prevalence, Non-cystic fibrosis bronchiectasis, Epidemiology

## Abstract

**Background:**

Data on the prevalence of and factors associated with nontuberculous mycobacteria (NTM) in patients with non–cystic fibrosis (CF) bronchiectasis are limited. Our aim was to determine the prevalence and factors associated with isolation of NTM in this population.

**Methods:**

We performed a multicenter observational study of historical cohorts comprising consecutive patients with non-CF bronchiectasis and at least 2 sputum samples cultured for mycobacteria over a period of 5 years.

**Results:**

The study population included 218 adult patients (61.9 % women) with a mean (SD) age of 55.7 (16) years and a mean (SD) of 5.1 (3.3) cultures/patient. NTM was isolated from sputum in 18 patients (8.3 %). Of these, 5 patients (28 %) met the American Thoracic Society criteria for NTM disease. *Mycobacterium avium complex* was the most frequently isolated microorganism (9 patients, 4.1 %). The variables independently associated with isolation of NTM were FVC ≥ 75 % predicted (OR, 4.84; 95 % CI 1.47 to 15.9; *p* < 0.05), age ≥ 50 years (OR, 4.74; 95 % CI 1.25 to 17.97; *p* < 0.05), and body mass index (BMI) ≤ 23 kg/m^2^ (OR, 2.97; 95 % CI 1.03-8.58; *p* < 0.05). Patients with these three characteristics had a 40 % probability of having at least one isolation of NMT.

**Conclusions:**

A significant number of patients with non-CF bronchiectasis are positive for the isolation of NTM. *M. avium complex* is the most frequently isolated mycobacteria. FVC ≥ 75 % predicted, age ≥ 50 years, and a BMI ≤ 23 kg/m^2^ were independently associated with the presence of NTM in patients with non-CF bronchiectasis.

## Background

Bronchiectasis is a chronic respiratory condition characterized by cough, sputum production, and increased susceptibility to lower respiratory tract infections. Impairment of mucus clearance facilitates recurrent respiratory infections [1]. While the lower respiratory tract is normally sterile, conditions such as bronchiectasis enable colonization by microbes such as bacteria. However, the airways of patients with bronchiectasis are also chronically colonized or infected by microorganisms, such as yeasts, filamentous fungi, and nontuberculous mycobacteria (NTM) [2]. NTM are ubiquitous in water and soil. Most cases of human infection are caused by NTM from the environment [3].

The incidence of lung colonization and infection due to NTM in patients with cystic fibrosis (CF) and non-CF bronchiectasis is increasing worldwide [4]. Since NTM diseases are not notifiable, epidemiological data are not readily available. The organism most frequently associated with these infections is *Mycobacterium avium complex* [5–7]. Isolation of pulmonary NTM does not necessarily indicate advanced infection or “disease,” since NTM may be present in respiratory tract secretions with no evident signs of illness (“colonization”). In pre-existing lung disease, especially in patients who experience frequent exacerbations (eg, those with bronchiectasis), defining clinical and radiological criteria that are specific for NTM diseases is more difficult.

Most published data on the prevalence of and factors associated with NTM in bronchiectasis are obtained from patients with CF. A recent meta-analysis by Chu et al. showed that the overall prevalence of NTM was 9.3 % in patients with non-CF bronchiectasis. Nevertheless, data on patients with non-CF bronchiectasis are limited, and most studies in this population have small samples [7]. Therefore, the aims of the present study were to determine the prevalence of NTM in a multicentre cohort of consecutive adult patients with non-CF bronchiectasis and to determine factors that are independently associated with isolation of NTM.

## Methods

### Study design

We performed an observational study of historical cohorts from 4 Spanish teaching hospitals with multidisciplinary and standardized non-CF bronchiectasis outpatient clinics.

### Study population

The study population comprised 296 consecutive patients aged ≥ 18 years who had been diagnosed with non-CF bronchiectasis of widely varying causes and for whom radiological extension and clinical and functional impairment were confirmed. Patients had to have been followed for at least 5 years during the period 2002–2010 before they could be considered for inclusion in analysis.

Patients had to have at least 2 sputum samples cultured for mycobacteria while (in a clinically stable phase) during the 5 years after the diagnosis. According to the recommendations of the Spanish Society of Pulmonology and Thoracic Surgery, the causes ruled out in idiopathic bronchiectasis were as follows: immunodeficiency with evidence of defective antibody production, gastroesophageal reflux disease, allergic bronchopulmonary aspergillosis, mycobacterial infections prior to development or diagnosis of bronchiectasis, cystic fibrosis, primary ciliary dyskinesia, and α1-antitrypsin deficiency [8]. CF was ruled out by 2 negative sweat test results in patients with bronchiectasis of unknown cause or a clinical presentation compatible with CF [9]. The study was approved by the Ethics and Research Committee of each center (registration number of the coordinating center: 0088-89-2011).

### Diagnosis of bronchiectasis

Bronchiectasis was diagnosed based on a high-resolution computed tomography scan of the chest that was interpreted by radiologists experienced in respiratory disorders. Images were obtained in full inspiration (1-mm collimation and 10-mm intervals from the apex to the base of the lungs). The presence of bronchiectasis was confirmed based on the criteria published by Naidich et al. [10]. The extent of bronchiectasis was evaluated according to the number of lobes affected, with the lingula and middle lobe considered as independent lobes.

### Data collection

Data were collected from all clinically stable patients and included the following: age, gender, body mass index (BMI, kg/m^2^), etiology, smoking habit (pack-years), dyspnea according to the modified Medical Research Council scale, macroscopic appearance of sputum (percentage of patients with purulent sputum), type of bronchiectasis (cystic or noncystic), radiological findings (number of lobes affected by bronchiectasis), and spirometry findings (forced vital capacity [FVC] and forced expiratory volume in the first second [FEV_1_] as both absolute values and percent predicted). We also recorded hospitalizations secondary to severe exacerbations and the number of exacerbations. All variables were obtained within 6 months after the radiological diagnosis of bronchiectasis, except for hospitalizations and the number of exacerbations, which were obtained prospectively during the year after the radiological diagnosis. Long-term treatments (antibiotics, oral macrolides, and oral corticosteroids) taken for at least 1 year after the radiological diagnosis of bronchiectasis were also recorded.

One sputum sample was recovered every 6 months during a clinically stable phase and cultured for mycobacteria, bacteria, and fungi. Additional sputum cultures were obtained whenever considered necessary by the clinician.

A stable clinical situation was defined as the absence of clinical criteria of exacerbation and no antibiotics or corticosteroids in the preceding 4 weeks [11]. Exacerbation was defined as the acute onset and persistence of changes in sputum characteristics (increased volume, thicker consistency, greater purulence, and hemoptysis) and/or increased breathlessness unrelated to other causes [12].

### Microbiology

Each respiratory sample was collected under sterile conditions and processed immediately or conserved at 4 °C. Smears were stained using the auramine acid fast method and scanned with microscopy fluorescence light at × 200 and × 400. All respiratory samples were processed according to the Tacquet-Tison technique. Processed samples were inoculated on Lowenstein-Jensen, Coletsos, and liquid medium (VersaTREK, formerly ESPII, Difco, Detroit, Michigan, USA). Cultures were incubated at 37 °C for at least 8 weeks.

Classic phenotyping assays were used. A specific gene probe for detection of *M. avium complex* was first used in 1990 (Accuprobe, GenProbe Inc., San Diego, California, USA). In 2003, species identification was complemented by 16S rRNA gene sequence analysis.

Sputum samples underwent auramine acid-fast staining and examination for routine bacteriological examination simultaneously. Only sputum samples with more than 25 polymorphonuclear leukocytes and fewer than 10 squamous cells on Gram stain were considered valid samples and processed for bacterial culture. Bacterial chronic lung infection was defined as isolation of the same potentially pathogenic microorganism (PPM) after the diagnosis of bronchiectasis in >50 % of respiratory cultures during the 5-year study period [13].

### Statistical analysis

The statistical analysis was performed using SPSS, version 15.0 (SPSS, Chicago, Illinois, USA). All data were expressed as mean (SD) or median (IQR) for quantitative variables and as absolute values and percentages for qualitative variables. The normality of the distribution was assessed using the Kolmogorov-Smirnov test. Prevalence of isolation of NTM was defined as the percentage of patients with at least 1 positive culture for NTM during the study. Patients were divided into 2 groups: NTM-positive patients (those with at least 1 positive sputum culture that showed growth of mycobacteria at any visit) and NTM-negative patients. Depending on the distribution of the variables, the *t* test or Mann-Whitney test was used to compare 2 means, and the chi-square test (with Fisher exact test if necessary) was used to compare qualitative or dichotomous variables. Variables of clinical interest (according to the current literature or the researcher’s opinion) and those that presented statistically significant differences (*p* <0.1) in the univariate analysis were included as independent variables in a logistic regression model based on the backward stepwise technique (Wald test). In order to determine the probability of having at least 1 isolation of NTM in different patient profiles, ROC curves were used to dichotomize continuous variables of special clinical interest according to the researchers (age, FVC, and BMI). The cut-off points were those with the greatest capacity to discriminate between patients with and without NTM, as follows: FVC (%predicted, with a cut-off of 75 %), age (with a cut-off of 50 years), and BMI (with a cut-off point of 23 kg/m^2^).

The independent variables that were finally included were age ≥ 50 years, BMI ≤ 23 kg/m^2^, macroscopic appearance of the sputum (mucoid vs mucopurulent or purulent), FVC ≥ 75 %, and isolation of PPMs (apart from NTM) from the sputum. The odds ratio (OR) and confidence intervals (95 % CI) for the independent variables were also calculated. *P* values < 0.05 were considered statistically significant in the logistic regression analysis.

A figure was constructed to show the different patient profiles with their probabilities of having at least 1 isolation of NTM depending of the presence or absence of the dichotomized variables independently associated with the isolation of NTM.

## Results

### Study population

Of the 296 patients screened, 218 met the eligibility criteria and were included in the study. The mean (SD) age of patients was 55.7 (16) years and 61.9 % were women. FVC was 66.8 ± 21.6 % predicted, FEV_1_ 62.6 ± 26.1 % predicted, and BMI 24.8 (4.6) kg/m^2^. All patients had at least 2 sputum samples cultured for mycobacteria during the 5-year study, with a mean of 5.1 (3.3) cultures/patient. The most frequent known aetiology of bronchiectasis was postinfectious causes (35.8 %), followed by chronic obstructive pulmonary disease (13.8 %), systemic diseases (12.8 %), immunodeficiency (2.3 %), and primary ciliary dyskinesia (3.2 %). The aetiology was unknown in 29.8 % of cases. Cystic bronchiectasis was detected in 56 patients (25.7 %).

### Prevalence of mycobacterial species

**Fig. 1:**
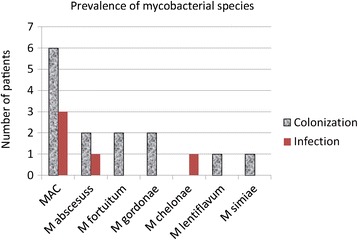
Number of patients with positive non-tuberculous mycobacterial cultures (NTM), grouped by mycobacterial species. Patients who met American Thoracic Society/Infectious Disease Society of America (ATS/IDSA) microbiologic criteria for NTM disease are represented in the shaded bars and those who did not meet ATS/IDSA criteria are represented in solid bars. The patient with *Mycobacterium simiae* also had *M. avium* complex (MAC)

NTM was isolated at least once from sputum in 18 patients (8.3 %) and *M. tuberculosis* from 2 patients. *M. avium complex* was the most frequently isolated microorganism (9 patients, 4.1 %). Other mycobacterial species were recovered from 9 patients (4.1 %) and included *M. abscessus* (3 patients), *M. fortuitum* (2), *M. gordonae* (2), *M. chelonae* (1), *M. lentiflavum* (1), and *M. simiae* (1). One patient had *M. simiae* and *M. avium complex*. Smears were positive in 7 of the 218 eligible patients (3.2 %). Of the 18 patients in whom NTM was isolated, only 6 (33 %) were smear-positive, and 5 (28 %) met the ATS/IDSA criteria for NTM lung disease [14] (3 *M. avium complex*, 1 *M. abscessus*, 1 *M. chelonae*) (Fig. 1).

### Cohort characteristics and factors associated with isolation and disease caused by NTM

When we compared the 5 patients who met the ATS/IDSA criteria for NTM lung disease with the remaining patients (213), we found that the 5 patients were women. With respect to the remainder of the study population, these 5 patients were less likely to present cystic bronchiectasis (0 vs 26 %, *p* = 0.001) or chronic bacterial colonization (20 % vs 59 %; *p* = 0.01) and had better functional test results (FEV1 80.5 % vs 62.1 % [*p* = 0.035] and FVC de 85 % vs 74 % (*p* = 0.0429). None of the 5 patients died during follow-up.

The characteristics of patients in the NTM-positive and NTM-negative groups are shown in Table 1. Univariate analysis revealed that patients with NTM were significantly older (64 vs 54.9 years; *p* < 0.05) and tended to be less frequently chronically infected with *Pseudomonas aeruginosa* (28 % vs. 44 %), *Haemophilus influenzae* (11 % vs. 23 %), and other PPMs (12 % vs. 45 %). In addition, *Aspergillus* was more frequently isolated than in culture-negative patients (39 % vs. 26 %), purulent sputum was less frequent (41 % vs. 62 %), and lung function was better.

**Table 1:** Patient characteristics for patients with and without Non-tuberculous mycobacteria (NTM) cultured from their sputum during the study period

Variable	NTM positive	NTM negative	*p*-value
	(*n* = 18)	(*n* = 200)	
Age, yr^a^	64 (13.3)	54.9 (15.9)	0.02
Age ≥ 50 years	15 (83.3 %)	126 (63 %)	0.06
Gender (% females)^b^	78 %	60 %	0.2
BMI, kg/m^2^ ^a^	23.5 (4.8)	24.9 (4.6)	0.24
BMI ≤ 23 kg/m^2^ ^b^	11 (61.1 %)	65 (32.5 %)	0.034
Idiopathic	5 (27.8 %)	60 (30 %)	0.79
Post-infection	6 (33.3 %)	72 (36 %)	
Systemic diseases	4 (22.2 %)	24 (12 %)	
Immunodeficiency	1 (5.6 %)	4 (2 %)	
COPD	2 (11.1 %)	28 (14 %)	
Ciliary dyskinesia	0	7 (3.3 %)	
Other	0	5 (2.5 %)	
Smoking history (pack-years)^c^	22.2 (33)	11.6 (25.6)	0.2
Dyspnea (mMRC)	1.29 (1.3)	1.33 (1.13)	0.8
Macroscopic appearance of sputum (muco-purulent or purulent)^a^	7 (39 %)	125 (62.5 %)	0.045
Cystic bronchiectasis^b^	4 (22 %)	52 (26 %)	0.7
Number of affected lobes^a^	2.9 (1.3)	2.6 (1.2)	0.2
FVC, % predicted^a^	82.5 (23)	73.5 (24)	0.09
FVC ≥ 75 % predicted^b^	14 (77.8 %)	94 (47 %)	0.011
FEV_1_, % predicted^a^	72.3 (26)	63 (25)	0.15
Chronic *P. aeruginosa* infection^b^	5 (28 %)	88 (44 %)	0.1
Chronic *H. influenzae* infection^b^	2 (11 %)	46 (23 %)	0.1
Chronic bacterial infection, other PPMs^b^	2 (12 %)	90 (45 %)	0.05
Isolation of *Aspergillus* spp^b^	7 (39 %)	52 (26 %)	0.2
Macrolides (chronic use)^b^	3 (17 %)	22 (11 %)	0.4
Antibiotics (chronic use)^b^	4 (22 %)	70 (35 %)	0.2
Systemic steroids (chronic use)	1 (6 %)	8 (4 %)	0.6
Hospital admissions^c^	0.5 (0.7)	0.7 (1.1)	0.5
Exacerbations^c^	2.18 (1.7)	3 (2.1)	0.2

*BMI* body mass index, *COPD* chronic obstructive lung disease, *mMRC* modified Medical Research Council, *PPMs* potentially pathogenic microorganisms

^a^Expressed as mean (SD)

^b^Expressed as frequency, number (%)

^c^Expressed as median (IQR)

Table 2 shows the results of the fully adjusted logistic regression analysis. Patients with isolation of NTM were nearly 5 times as likely to be aged ≥ 50 years and have an FVC > 75 % predicted value and nearly 3 times as likely to have a BMI ≤ 23 kg/m^2^.

**Table 2:** Logistic regression showing the factors independently associated with the presence of Non-tuberculous mycobacteria in the sputum of patients with non-cystic fibrosis bronchiectasis

Variable	B	OR	95 % IC	*p*
FVC ≥ 75 % predicted	1.58	4.84	1.47–15.9	0.009
Age ≥ 50 years	1.56	4.74	1.25–17.97	0.022
BMI ≤ 23 kg/m^2^	1.09	2.97	1.03–8.58	0.045

Full adjusted model include: FVC ≥ 75 % predicted, age ≥ 50 years, body mass index (BMI) ≤ 23 kg/m^2^, presence of chronic bacterial infection by potentially pathogenic microorganisms and macroscopic appearance of the sputum (mucous vs mucopurulent or purulent)

**Fig. 2:**
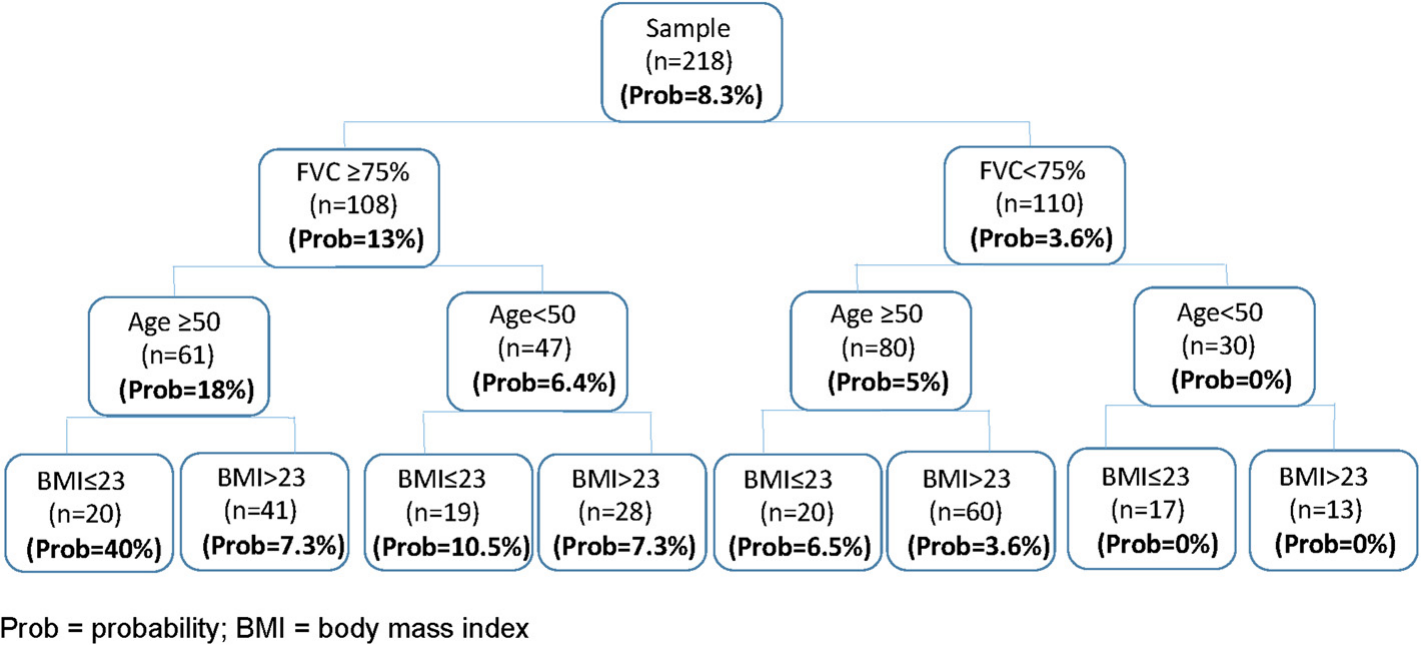
Probability of the isolation of non-tuberculous mycobacteria in patients with non-cystic fibrosis bronchiectasis by patients characteristics. Prob = probability; BMI = body mass index

Figure 2 shows a patient profile probability tree for the isolation of NTM at least once depending on the 3 variables independently related to isolation of NTM. The patient profile with the greatest probability (40 %) of isolation of NTM comprised all 3 characteristics (FVC predicted ≥ 75 %, age ≥ 50 years and BMI ≤ 23 kg/m^2^); the patient profile with the lowest probability of isolation of NTM (0 %) in our series comprised FVC < 75 % predicted and age < 50 years.

## Discussion

Our study indicates that the prevalence of NTM isolates is high in patients with non-CF bronchiectasis and that *M. avium complex* is the most commonly isolated microorganism. Based on our results, FVC ≥ 75 % predicted, age ≥ 50 years, and BMI ≤ 23 kg/m^2^ were independently associated with the presence of NTM at least once in patients with non-CF bronchiectasis. Furthermore, these patients tended to be less frequently chronically infected by *P. aeruginosa* and *H. influenzae.*

In the present study, the prevalence of NTM was 8.3 %, which is consistent with values reported previously in adult patients with non-CF bronchiectasis (2 to 37 %) [15–17]. Consistent with previous findings [7], *M. avium complex* was the most frequently isolated pathogen. Again, as indicated by other authors [15, 16], it is notable that smears were positive in only 33 % of patients with positive cultures.

Data on the natural history, prognosis, and associated factors of adult patients with non-CF bronchiectasis and coexisting NTM infection are limited, and most studies were conducted in CF patients with bronchiectasis. In both CF and non-CF patients, age is the only risk factor clearly associated with NTM [17–19]. However, in the present study, we found that other factors were associated with isolation of NTM. In this sense, NTM-positive patients tended to have better lung function, lower BMI, lower frequency of chronic *P. aeruginosa* infection, and a higher frequency of *Aspergillus*. NTM-positive patients also had fewer exacerbations and less frequently took chronic antibiotic therapy. A previous report found that NTM were more likely to be isolated in female patients with low BMI [17].

We found that age ≥ 50 years and FVC ≥ 75 % predicted were independently associated with isolation of NTM. The association between NTM and significantly older age in patients with mild disease may be a phenomenon of repeated, prolonged environmental exposure that is not related to the severity of pulmonary disease. Olivier et al. suggest that patients with more severe lung disease could die before having sufficient exposure time to acquire and retain mycobacteria [6].

Although several published reports on CF and non-CF bronchiectasis patients found that lung function and colonization by *P. aeruginosa* were positively, negatively, or inconsequentially associated with the development of NTM [15–17, 20–23], a large American multicenter study in CF patients showed that patients who were culture-positive for NTM were older, had milder disease, and were more likely to be colonized with *Staphylococcus aureus* but not with *P. aeruginosa* [6]. In the present study, NTM-positive patients tended to have better lung function and a lower frequency of chronic *P. aeruginosa* infection. *Aspergillus* was also more frequently isolated in this group. In fact, the factor most associated with the presence of bronchiectasis was mild functional impairment (FVC ≥ 75 % predicted). Patients with mild functional impairment presented a greater prevalence of NTM (13 %), making this variable the best predictor of isolation of NTM. Given that *P. aeruginosa* infection has been associated with worse pulmonary function and poor prognosis [24, 25], NTM-positive patients in our study, who were less commonly infected by *P. aeruginosa*, should have less impaired pulmonary function and a longer life expectancy. However, we cannot rule out the possibility that this association results from the difficulty in recovering mycobacteria from sputum with *P. aeruginosa* despite decontamination procedures [6, 26].

Although no clear association between NTM and *Aspergillus* spp has been demonstrated, several investigators have reported that NTM infection is associated with *Aspergillus*-related diseases in patients with non-CF bronchiectasis [18] and with higher rates of *A. fumigatus* culture in patients with CF [21, 23, 27] than in NTM-negative patients. Given that airway colonization by filamentous fungi has been associated with older age in CF patients with bronchiectasis [28, 29], perhaps the more frequent isolation of *Aspergillus* could be explained by the older age of NTM-positive patients in our study.

One of the key strengths of the present study is the very large cohort of patients with multiple sputum samples during the 5-year follow-up and the fact that we were able to construct an individual probability tree for the presence of bronchiectasis depending on the characteristics of the patient. Also noteworthy is the association with better pulmonary function and a trend towards an association with lower frequency of chronic infection by *P. aeruginosa.* Furthermore, in other studies on bronchiectasis and NTM, the study population was entered from a single tertiary referral center, while ours is the only multicenter study with a large patient population.

In addition to the logical restraints inherent to a retrospective design, our study is limited by the fact that the only factors we found to be clearly associated with isolation of NTM were age, lung function, and BMI. This finding may have been due to underpowering, since only 18 patients were NTM-positive. Furthermore, since we did not perform bronchoscopy, we could have underestimated the prevalence of NTM.

## Conclusions

In conclusion, we report an 8.3 % prevalence of NTM (2.3 % meeting ATS/IDS microbiological criteria for infection) in the sputum of screened non-CF patients with bronchiectasis in specialist clinics. *M. avium complex* appears to be the most common pulmonary NTM in this group of patients. Patients who are culture-positive for NTM are older and have a lower BMI and milder disease in functional terms. They also tend to have less frequent chronic infection by *P. aeruginosa* and more frequent isolation of *A. fumigatus* than culture-negative patients. Larger sample sizes will enable us to better evaluate the extent of the association of bronchiectasis with NTM, assess the clinical impact of the disease, and identify factors that are clearly associated with NTM in non-CF patients with bronchiectasis.

## Abbreviations

BMI: Body mass index
CF: Cystic fibrosis
NTM: Nontuberculous mycobacteria
PPMs: Potential pathogenic microorganisms
